# Nanogenomic synergy in Diabetes care: precision therapeutics for patients with multiple long-term conditions

**DOI:** 10.3389/fendo.2026.1767858

**Published:** 2026-02-10

**Authors:** Alok Raghav, Hamid Ashraf, Jamal Ahmad

**Affiliations:** 1 Adjunct Faculty, University Centre for Research & Development (UCRD), Chandigarh University, Chandigarh, India; 2 Adjunct Faculty, Centre for Global Health Research, Saveetha Medical College and Hospital, Chennai, India; 3 Rajiv Gandhi Centre for Diabetes and Endocrinology, J.N Medical College, Aligarh Muslim University, Aligarh, India; 4 Former Professor, Rajiv Gandhi Centre for Diabetes and Endocrinology, J.N. Medical College, Aligarh Muslim University, Aligarh, India

**Keywords:** diabetes mellitus, genetics, multiple long term complications, nanogenomics, precision medicine

## Abstract

Diabetes mellitus presents complex constellation of multiple long-term conditions (MLTCs) including cardiovascular, renal, metabolic, and neuroinflammatory disorders. This leads to clinical heterogeneity, polypharmacy, therapeutic disinterest, adverse outcomes, societal challenges, and glycaemic control management strategies. Precision medicine-based approach address this intricacy through alignment of therapeutic interventions with consideration of biological profiles. In this, nanogenomic synergy is a breakthrough, that gives detailed insight of genomic and epigenomics in context to advanced nanotechnology-based drug delivery system for personalized diabetes care. Genomics and transcriptomic profiling approach stratified the patients based on molecular drivers of diabetes and treatment responses, whilst nanotechnology facilitated the targeted, controlled and tissue specific systemic effect with minimal toxicity and off-target effects. This mini review will present the current evidence and understanding of clinical and biological obstacles in management of MLTC associated with diabetes mellitus and investigate the role of nano genomics through shard pathogenic pathways across comorbid conditions. Many key evidence nanotechnology platforms for genomic guidance in therapeutics were also covered. A nanogenomic synergy is a very innovative concept that could potentially break through one-size-fits-all approaches by allowing the redefinition of diabetic care for people living with multiple long-term diseases. This is achieved through precision, innovation, and a more encompassing approach to care.

## Introduction

Diabetes mellitus represents a major global health challenge and is increasingly encountered not as an isolated metabolic disorder but as part of a broader spectrum of multiple long-term conditions, including cardiovascular disease, chronic kidney disease, obesity, neurocognitive impairment, and chronic inflammatory syndromes (1, 2). Previously published literature showed multi-morbidity complications associated with diabetes mellitus (3–7). Diabetes mellitus in conjunction with multiple long-term conditions is associated with increased morbidity and mortality, as well as substantially higher healthcare utilization and costs related to disease management (8). Traditionally, established approaches to diabetes management have been largely reductionist, emphasizing glycaemic control through standardized treatment algorithms that inadequately account for biological heterogeneity, shared molecular pathways, and the complex interactions between coexisting diseases and medications in the setting of multimorbidity. This has contributed to polypharmacy, suboptimal clinical outcomes, and therapeutic inertia, particularly among older adults and socioeconomically disadvantaged populations. In contrast, precision medicine represents an advanced paradigm that seeks to individualize therapeutic strategies by integrating each patient’s unique biological, genetic, and environmental characteristics, thereby offering a more effective approach to the management of complex disease states. In this cutting-edge setting, nanogenomic synergy is a new field that is being actively investigated with the goal of revolutionising the way diabetes is currently managed in the presence of MLTCs by utilising novel drug delivery systems based on nanotechnology for genomic and epigenomic profiling (9). With additional opportunities for patient categorisation and therapy response prediction through genetic sequencing, this state-of-the-art technology offers the possibility of selective, regulated, and cell-specific therapeutic delivery. While promoting symptomatic alleviation, this intersection of several fields of study presents opportunities for customising the molecular pathophysiologic events that cause such disorders. This brief review aims to present an up-to-date view of nanogenomic synergy regarding diabetes management, with a focus on the importance of this new field of study among the MLTC populations, which are distinguished by their complex and varied states and need for novel therapeutic approaches that can fully address any circumstance.

Conventional diabetes mellitus management approach remain reductionist along with prioritizing glycemic control using standardized treatment algorithms extracted from randomized controlled trials (RCTs). These strategies do not appropriately consider the biologic heterogeneity introduced by the presence of the MLTCs, in which a variety of mechanisms, including chronic inflammation, insulin resistance, oxidative stress, endothelial dysfunction, and mitochondrial damage, contribute variably to the development and progression of a variety of diseases. The result in these instances would be an emphasis on a guided therapeutic approach that results in therapeutic inertia. Precision medicine is recently shown to be efficient in addressing this complexity. It addresses the complexity by using techniques networked around an individual biological identity uncovered using the assistance of genomic, epigenomic, and transcriptomic characteristics. Based on the above, there had been discoveries concerning the universal foundation on which the genetic characteristics related to the universal coexisting disease of the subject with diabetes and its coexisting diseases. Moreover, it is true that epigenomics pertaining to environmental elements and lifestyle had been revealed to be an added characteristic increasing the vulnerability to medication as well as the evolution of the diseases. Yet the utilization of the above knowledge using the knowledge developed on the molecular level up until this point had been stopped because of an important barrier presented by the absence of an efficient delivery mechanism competent in using the knowledge in preparing appropriate and safe medication concerning the subject with more than one coexisting disease.

Nanotechnology has an innovative application to bridge this translational gap. The use of advanced nanocarriers has the potential for controlled and tissue-specific biologically multifunctional delivery of small molecules, nucleic acid, and gene-editing therapies, with the aim of minimizing systemic side effects. The integration of nanotechnology and genomic and epigenomic profiling can lead to the strategic formulation of drugs based on molecular profiling of individual patients, irrespective of disease type. This integration of molecular stratification and nanotechnology forms the basis of the concept of nanogenomic synergies, where knowledge of the genome and epigenome translates to the strategic identification and modulation of nanotherapies targeting nanotechnology, based on the principle of precision medicine. Instead of devised strategies to use nanotechnology to treat diabetes and associated miscellaneous comorbidities, nanogenomic synergies focus on pathogenic routes, which are common to the MLTC groups. This work examines recent biological and translational advances to assess the relevance of integrating nano- and genomic technologies as a unified platform for diabetes management in the setting of multiple long-term conditions (MLTCs). The review first outlines the challenges posed by multimorbidity in the clinical management of diabetes. It then discusses the genomic and epigenetic heterogeneity underlying diabetes and its commonly associated conditions. Finally, contemporary developments in precision technologies are reviewed to highlight their convergence into nano-enabled strategies. Collectively, this mini-review provides a focused and up-to-date synthesis of functionally relevant advances supporting the translational potential of nano-genomic integration in complex diabetes care.

## Multiple long-term conditions in diabetes: a clinical and biological challenge

It is noteworthy that a substantial proportion of individuals with diabetes live with one or more multiple long-term conditions (MLTCs). According to epidemiological studies, over 60% of type 2 diabetic patients have one or more other chronic conditions, and a significant portion of patients have three or more additional chronic conditions (10). Due to common pathophysiological states including low-grade chronic inflammation, insulin resistance, endothelial damage, oxidative stress, or mitochondrial dysfunction, these disorders are likely to coexist in predictable, non-chance combinations. For example, the association between type 2 diabetes and cardiometabolic disorders or chronic kidney diseases shares certain pro-inflammatory and metabolic pathophysiological signals that drive the progression of their pathophysiology. In real-world clinical practice, the presence of multiple long-term conditions complicates the achievement of glycaemic targets by altering pharmacokinetics, increasing the risk of adverse drug–drug interactions, and introducing competing therapeutic priorities. It is also no uncommon for patients with type 2 diabetes to be on multiple medications for MLTCs, potentially escalating risk of either adverse interactions or other medication non-adherence or iatrogenic outcomes (11).

Moreover, therapeutic inertia, or delays in transitioning or maximizing therapy, is caused by the inability of clinicians to adequately balance risk versus benefit for multiple conditions. From a biological perspective, multiple long-term conditions amplify inter-individual variability in disease progression and therapeutic response, thereby limiting the effectiveness of population-based treatment strategies. Most evidence-based clinical guidelines are derived from randomized controlled trials focused on single diseases and routinely exclude patients with multimorbidity, which substantially reduces their applicability to real-world diabetic populations. This disparity underscores the need for treatment strategies tailored to multiple long-term conditions that explicitly account for individual biological variability and address the shared and disease-specific mechanisms underlying both diabetes and its associated complications. Nanogenomic approaches are unique in meeting this challenge by effectively targeting individual shared pathophysiologic defects with minimal need for systemic exposures of therapeutic agents, adequately meeting both biologic challenges of diabetic effects as well as challenges of MLTCs.

## Genomic and Epigenomic Determinants of Heterogeneity in Diabetes with MLTCs

Genome and epigenome diversity are one of the major factors that contribute to the heterogeneity observed in diabetes and other long-term diseases. Several genes that contribute to insulin secretion, insulin sensitivity, lipid metabolism, inflammation, and vascular function were found by Genome-Wide Analysis Studies of various disorders that often overlap with genes related to cardiovascular and renal diseases (12). Apart from the fixed differences in the genes, other factors such as DNA methylation, histone modification, and the regulation of non-coding RNAs help establish dynamic interactions between genes and their environments that influence the development, progression, and treatment of various diseases. Environmental factors such as dietary components, physical activity, psychosocial stress, environmental toxins, and early life exposures can provide permanent information to epigenetic systems that contribute to the emergence of multi-long-term conditions (MLTCs) characterized by clusters of various diseases (13). A transcript analysis study helps identify the molecular subtypes of diabetes with various levels of inflammation, metabolic impairment, and fibrosis that are related to varying risks of developing complications with low or high susceptibility to various treatments. Moreover, it is interesting to note that molecular signatures of such diseases are often altered by the presence of other diseases that contribute to the heterogeneity of such complex diseases. Pharmacogenomic studies on various antidiabetic medicines such as Metformin, Sulphonylureas, GLP-1 Receptor Agonists showed that genetic differences exist among various medicines that provide varying levels of resistance or susceptibility to various medications related to various adverse effects. In populations with multiple long-term conditions, various medicines’ effectiveness is hampered by the pathophysiological characteristics of concomitant diseases that provide varying levels of effectiveness with respective additional medications. A combination of genomic and epigenomic information helps subtype patients biologically who then remain an important step for identifying targets for effective therapeutic interventions. However, for effectively using the capability of the mentioned understanding of diseases, it is important that the respective health care systems would provide opportunities for context-sensitive interventions. A nanotechnology platform helps establish the capability of targeting genes that generate such diseases without any cytotoxic elements by developing nanosized medicine that would make targeted therapy feasible by establishing nanosized particles that could overcome any biological barriers for targeted gene therapy by establishing gene targets using nanotechnology platforms.

## Nanotechnology Platforms for precision Diabetes therapy

**Figure 1:**
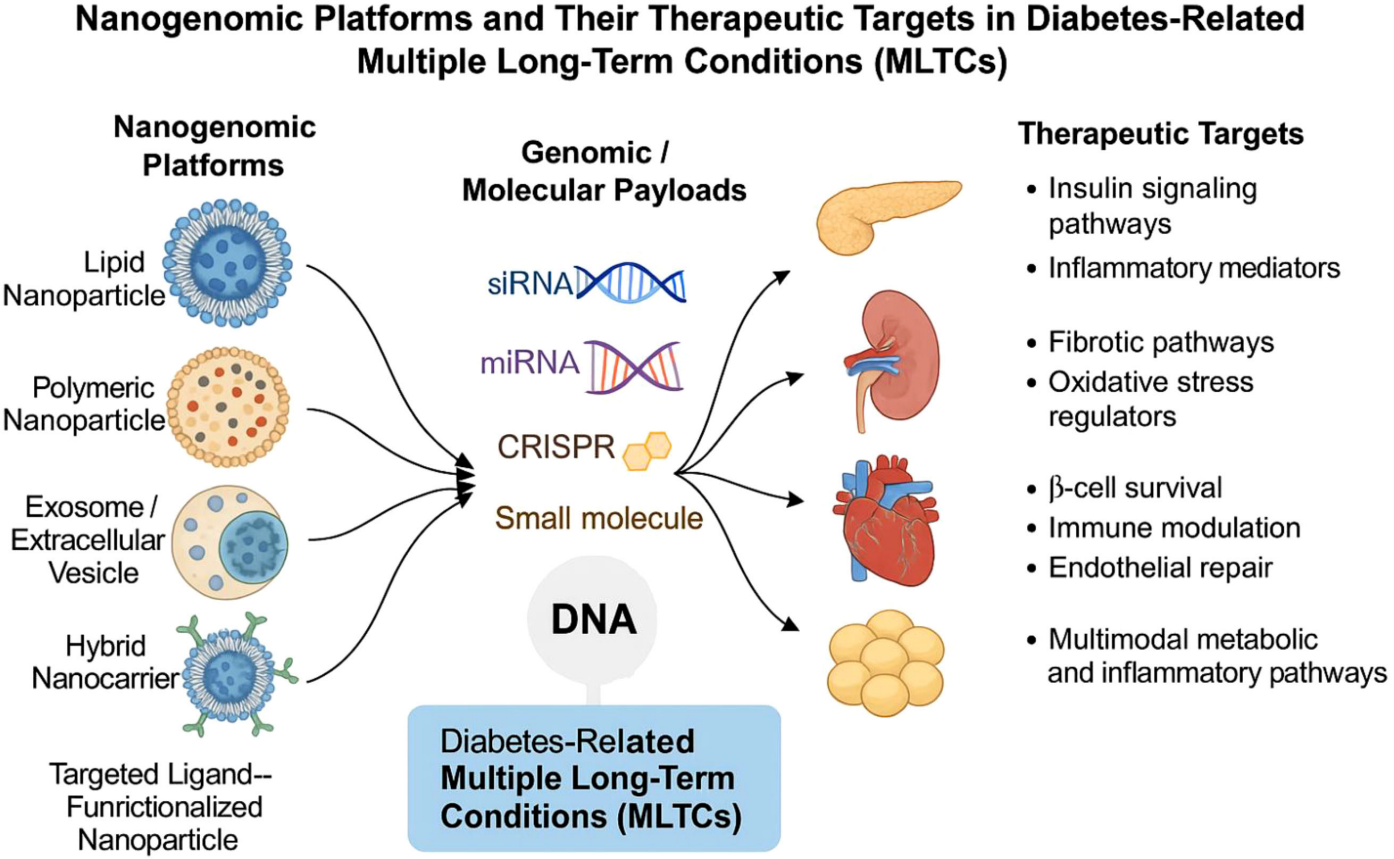
Nanogenomic platforms and their therapeutic targets in diabetes-related multiple long-term conditions (MLTCs).

Nanotechnology has heralded a new era in drug delivery by allowing the development of controlled, targeted, and multifunctional therapeutic systems to overcome many of the obstacles that traditionally came with pharmacotherapy. About the management of diabetes, different nanoparticle systems, including those of lipid nanoparticles, polymeric nanoparticles, inorganic nanostructures, and biologically derived exosomes, have so far shown great potential for improving therapeutic efficacy and safety. Lipid nanoparticles have gained tremendous popularity due to their biocompatibility, nucleic acid encapsulation capability, and ability to deliver small molecules intracellularly with sustained release. Polymeric nanoparticles possess tunable physicochemical properties that enable precise manipulation of the drug loading, release rate, and tissue targeting as shown in **Figure 1** (14). Because of their natural origin, innate targeting capabilities, and capacity to penetrate a variety of biological barriers, exosomes and other extracellular vesicles are among the most fascinating options. A variety of materials are combined to create hybrid nanocarriers, which improve stability, targeting effectiveness, and payload delivery.

These technologies have demonstrated potential for glucose-sensing insulin delivery systems, anti-inflammatory drug targeting, or organ-level metabolic pathophysiology modulation in the context of diabetes care. Notably, nanotechnology facilitates the simultaneous delivery of several medications, which makes combination therapy for several targets of different chronic diseases easier to administer. It may be possible to selectively target pancreatic β-cells, adipose tissues, liver, kidneys, or vascular endothelial tissues by using targeting ligands such peptides, antibodies, or transferrin linked to nanocarriers (15). Additionally, these nanocarriers may reduce unpleasant effects by minimising off-targeting pathophysiology, particularly in individuals with numerous comorbid illnesses. Thus, with the use of genetic stratification techniques, nanotechnology provides the justification for precision-mediated structural and functional targeting of diabetic therapy.

## Nanogenomic Synergy: Integrating Genomic Insights with Nanotherapeutic Design

The deliberate integration of tailored genomic data with targeted nanotherapeutic development within a precision feedback medicine model is the source of nanogenomic synergy. This includes the use of transcriptomic and genomic analysis to enhance the selection of molecular targets, pathway identification, and medication response prediction. The design and payload building of the nanoparticles, such as small molecule medications, siRNA, miRNA, antisense oligonucleotides, or CRISPR gene-editing elements, are determined by the acquired knowledge. For instance, nanocarriers of anti-inflammatory RNA therapeutic agents may be a suitable treatment for insulin resistance resulting from chronic inflammation, but tailored delivery of regenerative or protective agents will be necessary for β-cell pancreatic failure (16). Strategies to overcome unfavourable gene expression with customised epigenetic treatments are further informed by epigenomic data. Targeting specificity can be improved by designing the surface functionalisation of nanocarriers based on tissue-specific expression profiles of receptors that are revealed by genomic analysis.

Furthermore, dosage plans, release patterns, and carrier characteristics can be influenced by genetic markers of drug metabolism and toxicity in ways that reduce adverse effects. Most significantly, adaptive therapy in which a continuous molecular evaluation modifies the therapeutic action in real time is made possible by nanogenomic synergy. This is particularly important for the MLTC population, whose disease courses are nonlinear and impacted by a multitude of interrelated factors. Nanogenomic synergy is fundamentally different from traditional, one-size-fits-all technique since the treatment strategies can be tailored to everyone’s molecular biology. As a result, it offers a platform for completely personalised diabetes care. Though the term nanogenetics has not been formally institutionalized, the concept dates to the 1990s, when the first attempts were made to encapsulate plasmid DNA in liposomes to enhance transfection efficiency. Advances have quickened in the 2000s through polymeric nanocarriers and small interfering RNA (siRNA) therapeutics, culminating in the latest clinical breakthrough: lipid nanoparticles for delivering messenger RNA (mRNA) vaccines. Currently, nanogenetics is progressing toward clinical applications, with ongoing research exploring its potential for targeted gene modulation in metabolic diseases like diabetes. Meanwhile, genetic editing technologies like Clustered Regularly Interspaced Short Palindromic Repeats (CRISPR) transformed the accuracy with which genomes could be modified. According to this review, Nanogenetics is an interdisciplinary platform that combines nanoscale delivery systems with gene intervention to reprogram metabolic pathways. Nanogenetics, unlike classical gene therapy, is more focused on polygenic, environmentally sensitive disease biology, such as diabetes, where the metabolic network must be rewired. Such a definition positions nanogenetics as a paradigm independent of both traditional nanomedicine and genetic therapeutics. Nanogenetics differs from conventional diabetes and classical gene therapy in its dynamic and adaptive mode of action. While conventional therapies manage symptoms, gene therapy aims to achieve permanent correction, and nanogenetics enables controlled, reversible, and tissue-specific modulation of multiple genes via nanoscale carriers. This integration allows for feedback-responsive, temporally tunable regulation of metabolic pathways, offering a more precise and personalized approach to diabetes management (17).

## Therapeutic applications in diabetes with common MLTCs

By concentrating on both shared and disease-specific targets, nanogenomic methods have great potential for managing diabetes within the framework of conventional MLTCs. In cardiometabolic disorders, the use of nanoparticles for targeting anti-inflammatory, lipid-lowering compounds, or compounds for protecting endothelial cells could potentially slow the development of atherosclerosis, as well as improve glycemic outcomes (18). Moreover, for diabetic nephropathies, nanocarriers that target the kidney for antifibrotic or antioxidative compounds could potentially suppress the development of the nephropathies without developing either systemic side effects or other complications that could potentially hinder their efficiency. Talking about neuroendocrine, as well as any inflammation-related issues such as depression or cognitive impairments, nanocarriers that cross the blood-brain barrier could potentially suppress any gene targets of neuroinflammatory strategies inspired by the concept of metabolic holism. Moreover, obesity with insulin resistance is one major area that is of vital importance to be scrutinized, with nanotherapeutic approaches for targeting fat for any gene targets related to adipokines or inflammation would yield potentially similarly effective results (19). Finally, genomic stratification allows for selecting candidates for therapeutic approaches that is more effective with fewer off-target therapeutic effects of therapy. However, it is appreciable that it is vital to note that the applications of nanotechnology underscore convergence molecular targets of multiple diseases, in contrast to allocating multiple pathopharmacological states of pathophysiological differences with special emphasis on the complexities of MLTCs. Initially, any translation work endeavours for the previous applications clearly underscore the usefulness of this therapeutic approach with special emphasis on the importance of this therapeutic utility for complex diabetic states.

## Safety, ethical, and regulatory considerations

Although nanogenomic synergy is of great promise, it faces challenges of safety, ethics, and regulations, especially for the MLTC communities. Concerns about nanotoxicity remain valid since the issue of accumulation with respect to size and composition, as well as the immune system stimulation that could occur subsequently, remain valid. Additionally, there is a possibility of unintended interactions with genes other than the gene of interest, as well as heritability that could occur given that the cells are germline. Ethics relate to issues of equity of access for precision medicines, consent with respect to genomic analysis, genomic data privacy, or the prospect that new technology could potentially widen health disparities. There is also a lot of disparity with respect to nanotechnology and products related to the genome; it is for this reason that there is a challenge for their translation into clinical practice. Of ultimate importance is that the comprehensive preclinical assessment for safety, standardization of manufacturing processes, as well as flexibility for regulatory approaches, should be guaranteed for the beneficial application of the technology.

## Translational readiness and clinical integration

Translation of nanogenomic synergy from laboratory research into a clinical setting would need to overcome many scientific, logical, and systematic challenges. There is, however, serious evidence from the laboratory studies, while there are fewer clinical trials including the MLTC group, and reasons mainly include traditional exclusion criteria and conservative regulations. Cost-effectiveness and scalability should be considered, particularly in situations when the burden of numerous chronic illnesses is significant. For the genomic analysis to be implemented smoothly in the clinical setting, a multidisciplinary team approach and infrastructure development are essential. The variations in health care accessibility will also be considered in practical applications. Notwithstanding the difficulties, advancements in bioinformatics, manufacturing, and regulatory studies are making it more translation ready. It may also be improved by strategy coordination with precision public health initiatives.

## Future directions: toward systems-level precision care for MLTCs

If nanogenomic synergy is integrated into broad precision care models that incorporate AI, digital phenotyping, and multi-omics analysis, it will offer a highly promising future. AI-enhanced models can integrate intricate datasets to forecast illness trajectories and improve therapeutic strategies (20). Continuous monitoring a treatment plan that can be modified instantly is made possible by digital health technologies. Health economic assessments will be crucial for proving value and sustainability, especially in low- and middle-income nations where the prevalence of complex chronic illnesses is rising. Overall, nanogenomic synergy has the capability to largely alter diabetes management by amalgamating molecular precision with wider system innovations (9).

## Conclusion

Nanogenomic synergy is a transformational approach to managing diabetes and the growing number of people with several chronic conditions, in whom the complexity and heterogeneity of these diseases render traditional approaches ineffective. It refers to specific, adaptive, and pathway-oriented treatments tailored to a person’s biological profile through the bringing together of genomic information and cutting-edge nanotechnology platforms. While issues of safety, regulation, and practical application continue, the integration of precision medicine, nanotechnology, and innovation at the level of systems presents a particularly attractive avenue for reforming diabetes care in the context of multimorbidity.
